# Human Islet

**DOI:** 10.1111/cpr.70180

**Published:** 2026-04-10

**Authors:** Jiaqi Zou, Shen Li, Hongxing Fu, Mei Zhang, Anbin Hu, Xiaohui Tian, Yuanyuan Du, Lingling Wei, Xuchun Chen, Jie Gao, Rui Liang, Xinshun Feng, Yang Li, Ying Wang, Xiangheng Cai, Peng Sun, Xuejie Ding, Boya Zhang, Tengli Liu, Na Liu, Jie Hao, Lei Wang, Hongling Zhao, Jiani Cao, Aijin Ma, Lijun Zhu, Qiyuan Li, Qiang Wei, Xiaoming Ding, Ying Cheng, Tongbiao Zhao, Shusen Wang

**Affiliations:** ^1^ Tianjin First Central Hospital of Tianjin Tianjin China; ^2^ The Central Hospital of Dalian Dalian China; ^3^ The Second Affiliated Hospital of Zhejiang University Hangzhou China; ^4^ The Jiangsu Province Hospital Nanjing China; ^5^ The First Affiliated Hospital of Sun Yet‐Sen University Guangzhou China; ^6^ The First Affiliated Hospital of Xi'an Jiaotong University Xi'an China; ^7^ Hangzhou Institute of Medicine Chinese Academy of Sciences Hangzhou China; ^8^ Beijing Luhe Hospital of Capital Medical University Beijing China; ^9^ The First Hospital of China Medical University Shenyang China; ^10^ State Key Laboratory of Biopharmaceutical Preparation and Delivery, Institute of Process Engineering Chinese Academy of Sciences Beijing China; ^11^ Beijing Institute for Stem Cell and Regenerative Medicine Beijing China; ^12^ Institute of Zoology, Chinese Academy of Sciences Beijing China; ^13^ Standard Committee, Chinese Society for Cell Biology Shanghai China; ^14^ National Institutes for Food and Drug Control Beijing China; ^15^ Beijing Technology and Business University Beijing China; ^16^ Institute of Scientific and Technical Information of China Beijing China; ^17^ Shenzhen Medical Academy of Research and Translation Shenzhen China; ^18^ Institute of Laboratory Animal Science, Chinese Academy of Medical Sciences, Peking Union Medical College Beijing China

The standard ‘Human Islet’ is jointly drafted and agreed upon by experts from the Standard Committee of Chinese Society for Cell Biology. This standard specifies the technical requirements, test methods, inspection rules, instructions for use, labeling, packaging, storage, transportation, and waste disposal procedures for human primary islets and is applicable to their quality control. It was originally released by the China Society for Cell Biology on 28 October 2024. We hope that the publication of these guidelines will promote the establishment, acceptance, and execution of proper protocols and accelerate the international standardization of human islet for applications.

## 1 Scope

This document specifies the technical requirements, test methods, inspection rules, instructions for use, labeling, packaging, storage, transportation requirements, and waste disposal requirements for human islets.

This standard is applicable for the preparation and quality control of human islets derived from primary pancreas tissue for research use.

This standard does not apply to the preparation and quality control of human islet for clinical therapy.

## 2 Normative References

The content of the following documents constitutes indispensable provisions of this standard through normative reference. For dated references, only the edition cited applies. For undated references, only the latest edition (including all amendments) applies.

*WS 293 Diagnosis for HIV/AIDS*.

*Pharmacopoeia of the People's Republic of China (2020 Edition, Volume 3)*.

*National Guide to Clinical Laboratory Procedures (4th Edition)*.

## 3 Terms and Definitions

The following terms and definitions apply to this document.

### 3.1 Human Islet

Endocrine cell clusters isolated and purified from pancreatic tissue include α‐cells that secrete glucagon, β‐cells that secrete insulin, as well as PP cells and δ‐cells.

### 3.2 Human Pancreas Perfusion

A procedure used in the isolation of human islets that involves injecting a collagenase solution into the main pancreatic duct to digest connective tissue and separate islets from exocrine tissue, facilitating their isolation for research or transplantation.

### 3.3 Human Islet Isolation

The process of obtaining islets from pancreatic tissue.

### 3.4 Human Pancreas Digestion

The process of using collagenase perfusion to separate islets from pancreatic exocrine tissue.

### 3.5 Human Islet Purification

The process of purifying islets from pancreatic exocrine tissue to obtain high‐purity islets.

### 3.6 Human Islet Culture

The process of culturing the obtained islets in a cell incubator, with conditions adjusted according to different purity.

## 4 Abbreviations

The following abbreviations are applicable for this document.

BMI Body mass index

DNA Deoxyribonucleic Acid

EBV Epstein–Barr Virus

HbA1c Hemoglobin A1C

HCMV Human Cytomegalovirus

HIV Human Immunodeficiency Virus

IGF‐1 Insulin‐like growth factor‐1

SCID Severe Combined Immunodeficienc

UW The University of Wisconsin Solution

DMSO Dimethyl sulfoxide

DTZ Dithizone

FDA Fluorescent diacetate

HBV Hepatitis B Virus

HCV Hepatitis C Virus

IEQ Islet equivalent

PI Propidium iodide

STZ Streptozotocin

## 5 Technical Requirements

### 5.1 Raw Materials

#### 5.1.1 Basic Requirements

The requirements related to the donor pancreas source shall comply with the ‘Regulations on Human Organ Donation and Transplantation’ [1].

#### 5.1.2 Donor Pancreas Screening Criteria

##### 5.1.2.1 Exclusion Criteria

1. Type 1 or Type 2 diabetes;
2. Cancer [2];
3. AIDS, rabies, or risk factors for contracting above diseases [2];
4. Active tuberculosis [2];
5. Multidrug‐resistant bacterial infections [2];
6. Unexplained central nervous system infections [2];
7. Untreated or uncontrolled active infections and systemic infections [2];
8. Serious diseases of unknown etiology.

##### 5.1.2.2 Inclusion Criteria

The donor or pancreas shall meet these criteria:

1. Donor age between 18 and 60 years [2];
2. BMI ≥ 21 kg/m^2^ [2];
3. It is preferable to use pancreases from donors with HbA1c < 6.0% for islet transplantation [2]. Additionally, it is also necessary to assess the basic function of the islets according to the C‐peptide level of the donor.
4. Appearance assessment: The normal pancreatic length shall be 15–20 cm, with pale yellow color [2]. The head shall be flat, and the body and tail shall be slightly triangular prism shaped, with a texture slightly softer than that of kidneys;
5. Maximum 30 min warm ischemia time [2];
6. Other factors to consider: the cause of donor death, history of cardiac arrest, duration of hospitalization and ICU resuscitation time, hemodynamic status, use of vasoactive and hormonal medications, and other laboratory test results.

#### 5.1.3 Pancreas Harvesting

The liver, pancreas, duodenum, spleen, bilateral kidney and part of small intestine shall be removed by in situ perfusion and whole abdominal multi‐organ resection. When the donor pancreas is excised, the intact capsule of pancreas shall be preserved as far as possible, and blood vessels can be retained. The pancreas shall be fully perfused with organ preservation solution and rapidly cooled to 0°C–4°C to minimize the time of pancreatic hot ischemia [2]. The pancreas shall be carefully dissected and compression damage shall be avoided.

#### 5.1.4 Pancreatic transport, preservation and transfer

After the donor pancreas is completely obtained, it shall be stored in UW preservation solution at 0°C to 4°C [2]. The pancreas shall be packaged in a disposable aseptic bag with 2 to 3 layers of wrapping and placed in the specialized organ transport box. The temperature of the transport box shall be strictly monitored and recorded at 0°C–4°C during transporting the pancreas. The cold ischemia time of donor pancreas from harvesting to perfusing CIT Enzyme solution shall not exceed 12 h. Fill in the Human Pancreas Transfer Form according to the method in Supporting Information: Annex A.

### 5.2 Primary Quality Attributes

1. Human islet morphology.
  ○ The human islets should have a rounded surface, good refraction, and a diameter ranging from 50 μm to 500 μm.
2. Human islet number
  ○ The number of human islets is calculated in Islet Equivalent (IEQ), where one IEQ is defined as a spherical islet with a diameter of 150 μm.
3. Human islet viability
  ○ The human islets viability shall be 70%.
4. Purity of human islets.
  ○ A purity of ≥70% is defined as high‐purity islets, a purity between 40% and 69% is defined as medium‐purity islets, and a purity between 30% and 39% is defined as low‐purity islets.
5. Human islet function evaluation
  ○ a. Glucose‐Stimulated Insulin Release test (an in vitro test).
  ○ b. The Glucose‐Stimulated Insulin Release test defines the glucose stimulation index as the ratio of insulin secretion at high glucose levels to insulin secretion at low glucose levels. The islet function is considered qualified in vitro when the stimulation index is >1.
  ○ In Vivo Test
  ○ The function of the islets is assessed using an STZ‐induced diabetic nude mouse model with renal capsule transplantation of human islets. Blood glucose levels in the mice shall return to normal within 1 to 3 days after islet transplantation. If blood glucose does not drop to normal levels within 7 days, it indicates poor function of the transplanted islets. If blood glucose returns to normal post‐transplantation but then exceeds 13.8 mmol/L for three consecutive days, it indicates a loss of function of the transplanted islets.
6. Endotoxin
  ○ The endotoxin level in the islet suspension shall be below 5 EU/mL.
7. Microorganisms
  ○ Fungal, bacterial, mycoplasma, chlamydia, HIV, HBV, HCV, EBV, and HCMV shall be negative.

### 5.3 Process Control

1. Islet isolation
  - Cross‐contamination and confusion among different donors during islet isolation, tissues and cells shall be avoided, and risk countermeasures shall be established.
  - The islet culture after isolation shall specify the operation date, culture conditions, and the name or initials of the operator.
2. Islet isolation process and cell identification
  - The equipment, culture system, and operating procedures used for islet isolation shall be clearly defined, establishing a standardized and reproducible process. The specific operating procedures for islet isolation and culture shall comply with the requirements outlined in Supporting Information: Annex B. Record the Donor and Organ Procurement Organizations (OPO) information in Supporting Information: Annex B according to the Donor Basic Information Registration Form in Supporting Information: Annex I.
  - Islet morphology, yield, viability, and function shall be identified and documented.

## 6 Testing Methods

### 6.1 Islet Morphology

The test shall be conducted according to the method outlined in Supporting Information: Annex C.

### 6.2 Islet Number

The method in Supporting Information: Annex D shall be followed.

### 6.3 Viability of Human Islets

The method in Supporting Information: Annex E shall be followed.

### 6.4 Purity of Human Islets

The method in Supporting Information: Annex F shall be followed.

### 6.5 Functional Assessment of Human Islets

The in vitro functional assessment of human islets shall be conducted according to the method outlined in Supporting Information: Annex G.

The in vivo functional assessment of human islets shall be conducted according to the method outlined in Supporting Information: Annex H.

### 6.6 Endotoxin

The method ‘Bacterial and Endotoxin Test’ in the Pharmacopoeia of the People's Republic of China (2020 Edition, Volume 3) shall be followed.

### 6.7 Microorganisms

#### 6.7.1 Bacteria and Fungi

The test shall be conducted according to the ‘Sterility Test Method 1101’ in the Pharmacopoeia of the People's Republic of China (2020 Edition, Volume 3).

#### 6.7.2 Mycoplasma

The test shall be conducted according to the “Mycoplasma Test Method 3301” in the Pharmacopoeia of the People's Republic of China (2020 Edition, Volume 3).

#### 6.7.3 HIV

The test shall be conducted according to the ‘Nucleic Acid Method’ in WS 293.

#### 6.7.4 HBV

The test shall be conducted according to the ‘HBV Nucleic Acid Method’ in the National Clinical Laboratory Procedures (4th Edition).

#### 6.7.5 HCV

The test shall be conducted according to the ‘HCV Nucleic Acid Method’ in the National Clinical Laboratory Procedures (4th Edition).

#### 6.7.6 EBV

The test shall be conducted according to the ‘EBV Nucleic Acid Method’ in the National Clinical Laboratory Procedures (4th Edition).

#### 6.7.7 HCMV

The test shall be conducted according to the ‘HCMV Nucleic Acid Method' in the National Clinical Laboratory Procedures (4th Edition).

## 7 Inspection Rules

### 7.1 Sampling Method

1. Human islets produced from the same donor, same source, and same method are considered to be the same batch.
2. For human islets from the same batch, three samples shall be extracted at different purities (high purity, medium purity, low purity) for testing.

### 7.2 Quality Inspection and Release

1. Each batch of products shall be subject to the quality inspection before release, and inspection reports shall be attached.
2. The quality inspection items shall include all the attributes specified in Section 5.2.

### 7.3 Review Inspection

1. Review inspection shall be performed by professional cytological testing institutions or laboratories as necessary.

### 7.4 Decision Rules

1. Products that pass all requirements in Section 5.2 (excluding “In Vivo Test” In Vivo Functional Assessment) for the quality inspection for release are considered to be qualified. Products that fail to pass one or more requirements in Section 5.2 (excluding “In Vivo Test” In Vivo Functional Assessment) for the quality inspection for release are considered to be unqualified.
2. Products that pass all requirements in Section 5.2 (excluding “In Vivo Test” In Vivo Functional Assessment) for the quality review inspection are considered to be qualified. Products that fail to pass one or more requirements in Section 5.2 (excluding “In Vivo Test” In Vivo Functional Assessment) for the review inspection are considered to be unqualified.

## 8 Instruction for Usage

The instructions for usage shall include, but are not limited to:

- Product name;
- Islet batch number (Note: batch number from H001, the human islets isolated by the same donor source and the same method are an independent number);
- Islet equivalent number;
- The date of isolation and purification;
- Islet tissue volume;
- Storage conditions;
- Shipping conditions;
- Operation manual;
- Execution standard number;
- Manufacturing address;
- Contact information;
- Postal code;
- Matters that need attention.

## 9 Labels

The label shall include but not limited to:

- Product name;
- Islet batch number;
- Islet equivalent number;
- The date of isolation and purification;
- Islets tissue volume;
- Production date.

## 10 Package, Storage, and Transportation

### 10.1 Package

The appropriate materials and containers shall be selected to ensure the maintenance of the primary quality attributes of human islets.

### 10.2 Storage (Culture)

Products shall be stored at 22°C–37°C by culture. The time of islet culture used by transplantation is limited to 72 h.

### 10.3 Transportation

Human islets products shall be transported at 2°C–8°C.

## 11 Waste Disposal

1. Wastes generated during human islet production and testing shall follow the waste cell management documents, strictly implement the management standards and make detailed records.
2. Any disposal of unqualified cells, leftover cells for disposal or donations during the research and production of human islets shall be conducted properly in accordance with appropriate legal and ethical requirements.

Note: The Annexes are in the Supporting Information, which are available in the Supporting Information section.

## Author Contributions

Shusen Wang, Tongbiao Zhao, Ying Cheng, and Xiaoming Ding contributed to conception and design. Jiaqi Zou, Shen Li, Hongxing Fu, Mei Zhang, Anbin Hu, Xiaohui Tian, Yuanyuan Du, Lingling Wei, Xuchun Chen, Jie Gao, and Rui Liang drafted and revised the manuscript. Xinshun Feng, Yang Li, Ying Wang, Xiangheng Cai, Peng Sun, Xuejie Ding, Boya Zhang, Tengli Liu, Na Liu, Jie Hao, Lei Wang, Jiani Cao, Aijin Ma, Lijun Zhu, Qiyuan Li, and Qiang Wei critically read and revised the manuscript.

## Funding

This work was supported by National Key Research and Development Program of China (2024YFA1109002), National Natural Science Foundation of China (82470842), Tianjin Municipal Human Resources and Social Security Bureau (XB202011), funded by Tianjin Municipal Health Commission (TJYXZDXK‐3‐011B).

## Conflicts of Interest

The authors declare no conflicts of interest.

## Supporting information


**Data S1:** Supporting Information.

## Data Availability

The data that support the findings of this study are available from the corresponding author upon reasonable request.
