# Supplementary material for: Human Islet
Source: Cell Prolif. 2026 Apr 10;59(6):e70180. doi: 10.1111/cpr.70180 (PMC13241814; doi:10.1111/cpr.70180)
Supplement: Supplementary file 1 — Data S1: Supporting Information. [file CPR-59-e70180-s001.docx]

**ANNEX A**

**(NORMATIVE)**

HUMAN PANCREAS TRANSFER FORM

**A.1 The Human Pancreas Transfer Form is shown in Table A.1.**

| Table A.1 - Human Pancreas Transfer Form | | | |
| --- | --- | --- | --- |
| Project name |  | | |
| Islet ID number |  | | |
| Pancreas harvest Date |  | | |
| Transfer method (Preservation Solution) |  | Transfer temperature |  |

| Name of carrier |  | Date |  | |
| --- | --- | --- | --- | --- |
| List of accompanying Documents | Donor Information Registration Form (Source institution)  Yes🞎 Not🞎 | | | |
| Inspection items | 1. Is the appearance of the transport container intact  Yes🞎 No🞎  2.Appearance of the pancreatic organ packaging (clean, intact, without damage or leakage)  Yes🞎 No🞎  3. Was the transport temperature maintained between 2°C and 8°C during transit?  Yes🞎 No🞎 | | | |
| Name of receiver |  | Date | |  |
| Name of Reviewer |  | Date | |  |

**ANNEX B**

**(NORMATIVE)**

**STANDARD OPERATING PROTOCOL OF ISLET ISOLATION**

**B.1. INSTRUMENTS**

B.1.1 Cell counter.

B.1.2 Laboratory water bath.

B.1.3 Pancreas perfusion pump.

B.1.4 Digestion peristaltic pump.

B.1.5 COBE 2991.

B.1.6 Purity peristaltic pump.

B.1.7 Vacuum pump.

B.1.8 Temperature monitor.

B.1.9 Magnetic stir plate.

B.1.10 Refrigerated bench-top centrifuge.

B.1.11 Stereo microscope.

**B.2** **REAGENTS**

B.2.1 Pancreatic Wash Buffer: HBSS buffer, Amphotericin B solution, Iodophor solution, etc.

B.2.2 Perfusion Buffer: Liberase MTF C/F 2:3 GMP Grade, Heparin sodium, 1 M Hepes buffer, calcium chloride and HBSS buffer, etc.

B.2.3 Islet Wash Buffer: RPMI 1640 medium, Human serum albumin, Heparin sodium and Insulin, etc.

B.2.4 Islet collection solution: RPMI 1640 medium, Human serum albumin, Heparin sodium and Insulin, etc.

B.2.5 Islet purification stock solution: Ficoll 1.1, UW, etc.

B.2.6 Islet culture medium: CMRL1066 medium, Human serum albumin, Heparin sodium, IGF-1, etc.

B.2.7 Dithizone (DTZ) solution: DTZ powder, DMSO and HBSS buffer, etc.

**B.3** **CONSUMABLES**

B.3.1 Trimming kit: one tweezers, three accessories’ basins and one tray.

B.3.2 Perfusion kit: one tissue scissors, one tweezers, one tray, one needle holder, five hemostatic forceps and one knife handle.

B.3.3 Perfusion plate: one plate.

B.3.4 Ice tray: one.

B.3.5 Spiral heater: two.

B.3.6 Perfusion pipe: one kit.

B.3.7 Ricordi digestion tank: one kit.

B.3.8 Digestion pipes: one kit.

B.3.9 Density gradient beaker: one kit.

B.3.10 Purification pipes: one purification pipe A and one purification pipe B.

B.3.11 Cobe-bag: one set.

B.3.12 Collection bottles: three bottles.

B.3.13 Weighing bottle: one.

**B.4 PROCEDURE**

**B.4.1** **The pancreas acceptance and receipt**

B.4.1.1 Check the integrity of the package and confirm the donor information (serological test results, medical information, medical history);

B.4.1.2 The donor and Organ Procurement Organizations (OPO) information shall be recorded in the "Donor Basic Information Registration Form" according to the requirements outlined in Annex I.

**B.4.2 Pancreas preparation**

B.4.2.1 Using sterile technique, open the pancreas container and move the pancreas to a cold tray containing trimming solution;

B.4.2.2 Pour the organ preservation solution (UW) into the operating dish and surgically separate the duodenum and spleen;

B.4.2.3 Aseptically take at least 15 mL sample of the preservation solution for microbiology testing; 10 mL and 5 mL samples of the preservation solution were injected into the aerobic culture bottle and the anaerobic culture bottle respectively for bacterial culture Gram staining.

B.4.2.4 Prepare the Digestive Solution (the digestive enzymes used may vary from brand to brand and batch to batch, please refer to the product analysis/inspection report COA before use);

B.4.2.5 Sterilize pancreas surface with Iodophor sanitizer, Amphotericin B and HBSS buffer successively;

B.4.2.6 After excess tissue is trimmed from the pancreas, weigh the pancreas and record it in the batch record;

B.4.2.7 After the pancreas is cleaned of excess tissue, cut the pancreas to separate the head and tail, and cannulate the main pancreatic ducts using a small cannula.

B.4.2.8 Pancreatic tissue was examined before perfusion (including whether spleen and duodenum were attached, pancreatic injury, edema, perfusion, pancreatic surface fat and intra-pancreatic fat deposition, etc.).

**B.4.3 Pancreatic perfusion**

B.4.3.1 Perfuse the pancreas with the CIT Enzyme Solution;

B.4.3.2 Connect the perfusion tubing to the cannula and perfuse the pancreas for 5 minutes at 60 to 80 mmHg, followed by 10 minutes at 160 to 180 mmHg;

B.4.3.3 Cut the pancreas into 5 to 15 similar sized pieces of 2 to 4 cubic centimeter and place the pieces in a Ricordi digestion chamber.

**B.4.4 Pancreatic digestion (Phase 1 digestion)**

B.4.4.1 Preparation of islet wash solution and islet collection solution;

B.4.4.2 Add any remaining residual Enzyme Solution to the Ricordi Digestion Chamber for introduction into the digestion circuit. Add 1 to 2 mL of Dnase I (1 mL/ampoule, 4 mg/mL) to the Ricordi Digestion Chamber;

B.4.4.3 Start pumping the solution at a rate of 230 ± 20 mL/min to fill the system, and then add as much digestion solution to the Ricordi Digestion Chamber as needed to fill the system and to completely eliminate air from the circuit;

B.4.4.4 Shake the chamber gently for the first 5 minutes and then decrease the flow rate to 110 ± 20 mL/min after the temperature rises to 35℃. It takes approximately 3~5 minutes for the chamber to reach a target temperature of 36℃ to 38℃;

B.4.4.5 When tissue is observed in the circulatory digestive system, take a 1~2 mL sample of the digestion from the sampling port with a syringe every 1~2 minutes during the digestion, and observe the digest under a microscope. The results are recorded in batch record.

**B.4.5 Dilution and collection of islets (Phase 2 digestion)**

B.4.5.1 Adjust the temperature of the chamber to ≤ 30 ℃ during dilution and collection;

B.4.5.2 Collect the digest into the 1 L containers (islet collection solution was added in advance) (500mL, 400mL and 300mL collection solutions were added in the containers before cell collection);

B.4.5.3 When no islets are observed in the stained samples and little tissue remains in the chamber, discontinue the addition of media to the system, collect the media remaining in the system, and stop the circulation pump. Record the Dilution Stop Time at the end of the table below, and calculate and record the Total Dilution Time.

**B.4.6 Tissue recovery and washing**

B.4.6.1 As tissue is collected during dilution, transfer it to 225 mL conical tubes for the first four liters and centrifuge at 1000 RPM and 4 ˚C for 1 minute, to pellet the tissue;

B.4.6.2 Decant all of the supernatant and transfer pellets to 225 mL conical tubes containing 150 mL to 200 mL of Washing Solution (keep cold);

B.4.6.3 After centrifugation, discard the supernatant, combine the digestive products in the centrifuge tube, and re-suspend with the islet wash solution (centrifugation at 4˚C at 1000 RPM for 1minute);

B.4.6.4 After the washing is complete, the samples are resuspended by washing buffer within 1 mL~2 mL (4 mg/mL) of DNase. Take two 100 μL samples and count each sample once. Then centrifuge the final tube at 1000 RPM and 4 ˚C for 3 minutes.

B.4.6.5 Record the tissue volume and add 150mL UW solution (if the tissue volume is＞50mL, divide the tissue evenly into separate sterile 225 mL conical tubes and fill each to the 150 mL mark with additional UW Solution );

B.4.6.6 Re-suspended the islets, depending on the amount of tissue, with UW Solution.

**B.4.7 Islets purification**

B.4.7.1 COBE 2991 Preparation: After running tubing through pump, set COBE at 3000 rpm and pump to 450 mL/min, then run the "super-out" when the rotate speed to the maximum, run COBE for 3 minutes to wait for the next operation;

B.4.7.2 Assemble the COBE bag onto COBE cell processor according to institution’s procedure. Place clamps near the main line on all colored tubing except one line to be used for loading the COBE bag;

B.4.7.3 Place gradient-maker on magnetic stir plate and aseptically connect one end of purified tubing to gradient-maker and the other end to green tubing of the COBE bag;

B.4.7.4 Pour 110 mL of the High-Density Gradient (1.10 g/mL) into the left chamber of the gradient maker. Start to pump High Density Gradient (1.10 g/mL) into COBE bag. Once the entire 110 mL of High-Density Gradient (1.10g/mL) is loaded, remove excess air from the COBE bag by pressing Super-out while unclamping the red tubing. Press the Hold button once the Bottom Gradient has reached the T (junction of red/green tube). Re-clamp the red tubing line and press the Stop/Reset button;

B.4.7.5 Pour High Density Gradient in the left chamber (nearest the outlet) of the gradient maker, and Pour Low Density Gradient in the right chamber of gradient maker (away from outlet). Open and close the port between the two chambers just enough to fill the opening. Start the COBE and ensure that the centrifuge speed is at 1500 rpm, set pump to 0 mL/min.

B.4.7.6. Load the tissue with the pump. Gently swirl the beaker to keep the tissue well-suspended during the loading. As soon as the tissue is loaded, add 30 mL of additional washing solution to conical within the islet issue of pre-purification, load this rinse onto the COBE. After the last portion of the rinse has entered the COBE bag, stop the pump. Vent the system by carefully unclamping the red tubing. Re-clamp the tubing when liquid (capping solution) is approximately one inch above the ceramic seal. This is the start of centrifugation time.

B.4.7.7 Prepare 1 empty 225ml conical tube (labeled 0) and 6 225ml conical tubes (labeled 1, 2, 3, 4, 5, 6) added with 150 ml RPMI 1640 solution respectively;

B.4.7.8 Verify that the Superout Rate is set at 100 mL/min. After 5 minutes spin slowly remove the blue clamp on the green line and quickly press the Superout button. Collect the first 150 mL of effluent into the conical tube labeled “0” and 6 X 30 mL fractions into the numbered conical tubes each pre-filled with 150 mL RPMI 1640 Supplemented, then stop the COBE.

**B.5 Purity identification**

After the purification procedure is completed, gently mix centrifuge tubes 1～6, and sequentially take 1 mL of the sample from each tube to add into a 12-well culture plate. Perform DTZ staining and calculate the islet purity. A purity of ≥70% is defined as high-purity islets, a purity between 40% and 69% is defined as medium-purity islets, and a purity between 30% and 39% is defined as low-purity islets.

**ANNEX C**

**(NORMATIVE)**

**STANDARD DETECTION OF ISLET MORPHOLOGY**

**C.1 INSTRUMENTS**

C.1.1 Stereo microscope.

**C.2 REAGENTS**

C.2.1 The human islet culture medium contains the following main components: CMRL 1066 medium, human serum albumin, heparin sodium, and IGF-1. After preparation, the human islet culture medium should be filtered using a filter and stored at 2°C to 8°C.

C.2.2 The DTZ solution, with a concentration of 1 mg/mL, contains the following main components: DTZ, dimethyl sulfoxide (DMSO), and HBSS buffer. After preparation, the DTZ solution should be filtered using a 0.22 μm needle filter and stored at 2°C to 8°C in the dark. It should be prepared fresh for immediate use.

**C.3** **CONSUMABLES**

C.3.1 Cell culture dish

**C.4 TESTING PROTOCOL**

C.4.1 Preparing cell suspension

Harvest and suspend the islets with appropriate volume of HBSS Buffer.

C.4.2 DTZ staining

Evenly mix the DTZ with the cell suspension at a volume ratio of 1:1, stain for 1 min~2 min at room temperature.

C.4.3 The review of islet morphology record

C.4.3.1 Turn on the two switch buttons of the stereo microscope from left to right;

C.4.3.2 Place each sample stained with DTZ in a dish, Examine the islets sample (stained islets will appear red) using the 4×objective lens.

C.4.3.3 Observe immediately, describe the cell morphology after observation (such as whether it is clustered, whether there is debris, whether the edge is smooth, etc.), and take photos under a 4×objective. 20 IEQ islets were randomly selected from each field to observe and record the diameter, and determine whether the average diameter is between 50 μm and 500 μm across at least three fields.

C.4.3.4 Turn off the switch button of the ordinary optical microscope from right to left, and arrange the table.

**C.5 ASSESSMENT OF ISLET MORPHOLOGY**

Using Table C.1 as a guide for the assessment of islet morphology.

| Table C.1- Islets Morphology Scoring | | | |
| --- | --- | --- | --- |
|  | Count 1 | Count 2 | Average |
| %Trapped |  |  |  |
| %Fragment |  |  |  |
| Size | (0 0.5 1 1.5 2) | (0 0.5 1 1.5 2) |  |
| Fragment | (0 0.5 1 1.5 2) | (0 0.5 1 1.5 2) |  |
| Density | (0 0.5 1 1.5 2) | (0 0.5 1 1.5 2) |  |
| Margin | (0 0.5 1 1.5 2) | (0 0.5 1 1.5 2) |  |
| Morphology | (0 0.5 1 1.5 2) | (0 0.5 1 1.5 2) |  |
| Islet Morphology Score：________________ | | Name： | |

**ANNEX D**

**(NORMATIVE)**

**STANDARD QUANTIFICATION OF HUMAN ISLET**

**D.1** **INSTRUMENTS**

D.1.1 Stereo microscope

D.1.2 Cell counter

**D.2 REGENTS**

D.2.1 Human islet culture medium: 1066 Medium, Human Serum Albumin (HSA), Heparin Sodium, Insulin-like Growth Factor 1 (IGF-1), stored at 2 ℃ to 8 ℃.

D.2.2 DTZ Solution: DTZ (Dithizone), Dimethyl Sulfoxide (DMSO), HBSS Buffer,

After preparing the DTZ solution, it must be filtered using a 0.22μm needle filter and stored in a dark container at 2 ℃ to 8 ℃.

**D.3 CONSUMABLES**

D.3.1 Cell counting dish

**D.4 TESTING PROTOCOL**

D.4.1 Cell suspension preparation

Collect the islets by centrifuging and resuspend islets using HBSS buffer. Dilute the suspension to the appropriate concentration.

D.4.2 Cell staining

Add the 1mg/mL DTZ working solution to the islet suspension in a 1:1 ratio, mix thoroughly, and stain at room temperature for 1~2 minutes.

D.4.3 Cell counting

D.4.3.1 From left to right, sequentially turn on the two switch buttons of the stereo microscope.

D.4.3.2 Place the cell dish on the stage of the optical microscope, and gently move the islets to the center of the field of view. Locate the cells under the 4×objective lens (stained islets will appear red).

D.4.3.3 Use the standard scale in the eyepiece of the optical microscope to observe the size of the islets. Classify the islets into different diameter groups based on their size.

D.4.3.4 Use a cell counter to record the IEQ of islets in each diameter group.

D.4.3.5 After counting, sequentially turn off the switches of the optical microscope from right to left and clean the workspace.

**D.5 ANALYSIS OF RESULTS**

Islets with a diameter of 150μm are considered as one IEQ. The total IEQ of the sample is calculated by the following formula (Table D.1). Count the IPN and IEQ of islets with the dilution ratio.

**Table D.1 - Calculation of islet equivalents**

| Islet diameter range (μm) | Islets number (IPN) | Correction factor | Islet equivalent (IEQ) |
| --- | --- | --- | --- |
| 50~100 |  | 0.167 |  |
| 101~150 |  | 0.648 |  |
| 151~200 |  | 1.685 |  |
| 201~250 |  | 3.500 |  |
| 251~300 |  | 6.315 |  |
| 301~350 |  | 10.352 |  |
| 351~400 |  | 15.833 |  |
| ＞401 |  | 22.750 |  |
| Total islet number (IEQ) = Sum of IEQ values from each group × dilution factor. | | | |

Note: Islet count×conversion factor = IEQ for each group; the sum of the IEQ for each group gives the total IEQ of the islets obtained in this session. Islets with a diameter <50μm are not counted.

**ANNEX E**

**(NORMATIVE)**

**STANDARD DETECTION OF HUMAN ISLET VIABILITY**

**E.1 INSTRUMENTS**

E.1.1 Fluorescent microscope

E.1.2 Tabletop centrifuge

**E.2 REAGENTS**

E.2.1 FDA stock solution: Dissolve 1 mg of fluorescein diacetate (FDA) in 50 mL of acetone to obtain a 0.02 mg/mL (100×) stock solution. Aliquot the solution, protect it from light, and store at −20°C.

E.2.2 Dilute 10 μL the FDA stock solution into 1 mL PBS to prepare a 46 μM working solution. Use the working solution within 30 minutes. Note: Ensure complete dissolution of the dye during preparation to avoid clumping or particulate impurities during staining.

E.2.3 PI Stock solution: Weigh 1 mg propidium iodide (PI) and dissolve it into 1 mL DPBS to the stock solution (1 mg/mL, 100×). Aliquot the solution, protect it from light, and store at 2°C ~8°C.

E.2.4 PI Working solution: Dilute 10 μL the PI stock solution (1 mg/mL) into 1 mL of DPBS to prepare the 14.34 μM working solution. Use the working solution within 30 minutes.

E.2.5 Phosphate buffered saline (PBS): pH 7.4

**E.3 CONSUMABLES**

E.3.1 Cell culture dishes

**E.4 TESTING PROTOCOL**

E.4.1 Islet suspension preparation

Resuspend the islet samples with culture, transfer 100 μL of islet suspension into the culture dish with 900 μL of DPBS.

E.4.2 Islet staining

E.4.2.1 Quickly add 10 μL propidium iodide (PI) staining solution and 10 μL of fluorescein diacetate (FDA) staining solution to the islet suspension. Mix thoroughly using a micropipette to ensure even distribution. The final concentrations in the suspension are 0.46 μM FDA and 14.34 μM PI.

E.4.2.2. After staining for 1 to 2 minutes, the culture dish (30 mm×15 mm) was placed on a microscope slide, and review images using a fluorescence microscope.

E.4.3 Islet Viability Observation

E.4.3.1 Turn on the two switches of the fluorescence microscope from left to right.

E.4.3.2 Place the sample on the stage and locate the cells using a 4× objective lens.

E.4.3.3 Turn on the fluorescence laser emitter, adjust the excitation light pathway on the right side of the microscope stage, ensuring that the "blocker bar" does not obstruct the excitation light.

E.4.3.4 Capture: For each sample, randomly capture 3 fields of view. Take one image per field using a 4× objective lens. During the process, avoid capturing fluorescent impurities, as they may be mistakenly counted as cells during data analysis.

E.4.3.5 Turn off the fluorescence laser emitter (wait at least half an hour after turning it on before shutting it off). Then, sequentially turn off the regular optical microscope switches from right to left.

**E.5. ANALYSIS OF RESULTS**

E.5.1 Data processing

Randomly select three fields of view to assess the islet viability ratio (%), and calculate the average value (cells-stained green are live, while cells-stained red are dead).

E.5.2 Analysis of results

Eligibility criteria: The islet viability shall be ≥70%.

**ANNEX F**

**(NORMATIVE)**

**STANDARD DETECTION OF HUMAN ISLET PURITY**

**F.1 INSTRUMENTS AND EQUIPMENT**F.1.1 Stereomicroscope.

**F.2 REAGENTS**F.2.1 Human islet culture medium, with the following main components: CMRL 1066 medium, human serum albumin, heparin sodium, and IGF-1. The human islet culture medium should be filtered with a filter after preparation and stored at 2°C to 8°C.
F.2.2 DTZ solution, with a concentration of 1 mg/mL, containing DTZ, dimethyl sulfoxide (DMSO), and HBSS buffer. The DTZ solution should be filtered using a 0.22 μm needle filter after preparation and stored at 2°C to 8°C in the dark. It should be prepared fresh for immediate use.

**F.3 CONSUMABLES**F.3.1 12-well cell culture plate.

**F.4 TEST PROCEDURES**F.4.1 Cell Suspension Preparation
F.4.1.1 Collect purified samples into centrifuge tubes 1–6 according to purity from high to low, gently mix the islets, and sequentially take 1 mL samples from each tube to add into the 12-well plate.
F.4.1.2 Prepare a cell suspension with HBSS buffer and dilute to an appropriate concentration.

F.4.2 Cell Staining
F.4.2.1 Add 1:1 of the above 1 mg/mL DTZ working solution to the islet suspension, mix thoroughly, and stain for 1 to 2 minutes at room temperature.

F.4.3 Cell Purity Observation and Recording
F.4.3.1 Turn on the two switches of the stereomicroscope from left to right.
F.4.3.2 Place the 12-well cell culture plate on the optical microscope stage, gently move the cell clusters from high to low purity wells to the center of the field of view. Find the cells under the 4× objective lens (stained islets appear red).
F.4.3.3 Immediately observe and determine the islet purity based on the proportion of islets in the purified tissue.
F.4.3.4 After completing the purity observation, turn off the stereomicroscope switches from right to left and tidy up the workstation.

**F.5 RESULT DETERMINATION**Purity ≥ 70% is defined as high-purity islets, purity between 40% and 69% is defined as medium-purity islets, and purity between 30% and 39% is defined as low-purity islets.

**ANNEX G**

**(NORMATIVE)**

**ASSESSMENT OF HUMAN ISLET FUNCTION (IN VITRO TEST)**

**G.1 INSTRUMENTS**

G.1.1 Optical Microscope

G.1.2 High-speed Centrifuge

G.1.3 Ultrasonic Cell Disruptor

**G.2 REAGENTS**

G.2.1 Krebs buffer stock solution：

1.19 mM NaCl,

94 mM KCl,

50 mM CaCl_2_-2H_2_O

250mM NaHCO_3_

24mM KH_2_PO4

24mM MgSO_4_.7H_2_O,

10 mM HEPES,

0.5% BSA, pH=7.4

Note: CaCl₂·2H₂O shall be prepared separately. After adjusting the pH to 7.4, add it to the solution, as it may cause turbidity if added earlier.

G.2.2 Glucose: 50%.

G.2.3 Bovine Serum Albumin (BSA): purity≥ 98%.

G.2.4 RPMI-1640 medium: Store at 2°C~8°C.

G.2.5 Fetal Bovine Serum (FBS).

G.2.6 Phosphate buffered saline (PBS): pH 7.4

G.2.7 RIPA Lysis Buffer: Store at 2°C to 8°C, protected from light.

**G.3 CONSUMABLES**

G.3.1 6-well plates

G.3.2 12-well plates

G.3.3 EP tube

**G.4 TESTING PROTOCOL**

G.4.1 Testing preparation

Brief flow: 1.67 mM glucose (1 hour for equilibration), → 1.67 mM glucose (1 hour for sample collection), → 16.7 mM glucose (1 hour for sample collection), → Collect islets and perform ultrasonic lysis to extract total protein using 100 µL RIPA lysis buffer.

G.4.2 Testing operation

G.4.2.1 Krebs Buffer Stock Solution, High Glucose and Low Glucose Solution Preparation

G.4.2.2 Cell preparation

Remove human islets from the incubator, and manually select 10 IEQ of islets per well under a microscope according to the groupings. Place the islets in 1 mL of 1× PBS solution and wash the islets twice.

G.4.2.3 Pre-culture islet sample with low glucose

Add 1 mL of 1.67 mM glucose solution per well. Place 10 IEQ islets per well into the 1.67 mM low glucose solution. Pre-culture islet sample at 37°C with 5% CO₂ for 1 hour.

G.4.2.4 Low glucose incubation

For each group with three replicates, pre-add 1 mL of 1.67 mM glucose solution to each well, labeled as L1, L2, and L3 on the plate. Transfer the pre-cultured islets into the L1, L2, and L3 wells, ensuring the islets uniform in morphology and size between parallel wells. Place the dish under an inverted microscope with a 10× objective lens, review images. The imaging parameters shall be consistent for all wells. After imaging, place the dish in a 37°C, 5% CO₂ incubator, culture for 1 hour in low glucose conditions.

G.4.2.5 High glucose incubation

For three replicates, add 1 mL of high glucose solution to each well of a new 12-well plate, and label the wells as H1, H2, and H3. Transfer all the low glucose-incubated 10 IEQ islets into the corresponding H1, H2, and H3 wells. place the dish in 37°C, 5% CO₂ incubator for 1 hour under high glucose conditions.

G.4.2.6 Collect low-glucose supernatants

Collect the low glucose supernatant into 1.5 mL EP tubes and store at 2°C to 8°C.

G.4.2.7 Collect high-glucose supernatants

Collect the high glucose supernatant into 1.5 mL EP tubes and label them as H1, H2, and H3 accordingly. Store at 2°C to 8°C.

G.4.2.8 Detection of Insulin Levels

Detect insulin levels by ELISA (Mercodia). Read optical density at 450 nm and record the date.

**ANNEX H**

**(INFORMATIVE)**

**ASSESSMENT OF HUMAN ISLET FUNCTION (IN VIVO TEST)**

**H.1 INSTRUMENTS**

H.1.1 Stereo microscope

H.1.2 Glucometer

**H.2 REAGENTS**

H.2.1 Streptozotocin (STZ)

.2.2 Tribromoethanol

H.2.3 Amyl alcohol

H.2.4 HBSS (HEHPE-buffered saline solution)

H.2.5 Povidone-iodine

**H.3 CONSUMABLES**

H.3.1 PE50 Tube

H.3.2 Blood glucose test strips

**H.4 TESTING PROTOCOL**

H.4.1 Testing preparation

H.4.1.1 Mouse Husbandry and Handling

Mice shall be housed in VAF, SPF, or sterile facilities, receiving sterile food and water. All procedures, particularly with immunodeficient mice, shall follow institutional protocols for aseptic handling. Reagents and instruments must be sterilized prior to use to avoid contamination, ensuring the integrity of experimental results.

H.4.1.2 Mouse Blood Glucose Monitoring

(1) Blood Glucose Monitoring Timeline

Mice will undergo blood glucose monitoring for a minimum of 4 weeks after transplantation. If their blood glucose levels do not recover within 28 days post-transplant, glucose monitoring will be discontinued. If their blood glucose is normal at day 28, the mice will undergo left nephrectomy within the next 7 days. If these mice are diagnosed as diabetes again following nephrectomy, blood glucose monitoring will be terminated. If the mice maintain normal blood glucose levels post-nephrectomy, glucose monitoring will continue for an additional 14 days.

(2) Blood Glucose Monitoring Frequency, Recording, and Reporting

Blood glucose levels shall be recorded at least three times per week, with glucose concentration measured in mg/dL. Data shall be regularly reviewed in graphical form to detect trends or abnormalities.

H.4.1.3 Mouse Anesthesia

(1) Mice are anesthetized by intraperitoneal injection of 1.2% tribromoethanol (280 mg/kg).

(2) Please defer to individual facility and IACUC for most appropriate drug and route.

H.4.2 Testing operation

H.4.2.1 Preparation of chemically diabetic mice

(1) In a sterile manner, parenterally (IV or lP) administer facility-specific diabetogenic drug (i.e., streptozotocin or alloxan) at least 5～7 days prior to islet transplantation. Verify mice are normoglycemia prior to drug administration.

(2) After fasting for 4～6 hours, administer the drug parenterally (IP) with STZ (190 mg/kg).

(3) Maintain mice in sterile housing with access to adequate sterile food and water.

(4) Monitor blood glucose levels daily. Blood glucose levels > 19.4 mmol/L (350 mg/dL) for 2 consecutive days indicate that the model is successful.

H.4.2.2 Islet sample preparation

Prepare islets according to the method in Annex B. Calculate the islet equivalent using the procedure in Annex D.

H.4.2.3 Islet transplantation

Transplant 2000 IEQ human islets resuspended by equal volume HBSS buffer into diabetic nude mice induced STZ under renal capsular. Each group shall be at least 8 mice.

H.4.2.4 Mouse blood glucose monitoring

Monitor Non-fasting blood glucose levels at least 3 times per week for 4weeks.

H.4.2.5 Nephrectomy

If the diabetes has been reversed for at least 28 days after islet transplantation, nephrectomy will be performed to assure that the normoglycemia owe to transplanted islets.

**H.5 ANALYSIS OF RESULTS**

Under normal conditions, mouse blood glucose shall return to normal levels within 1 to 3 days post-islet transplantation. If blood glucose cannot recover normal levels within 7 days, it indicates dysfunction of the transplanted islets. If blood glucose level recovers normal post-transplant but exceeds 13.8 mmol/L for 3 consecutive days later, it indicates islet dysfunction.

**ANNEX I**

**(INFORMATIVE)**

DONOR BASIC INFORMATION REGISTRATION FORM

Birth date：_______/ _______ /_______ (Year/Month/Day) Age：_________

Height： _______cm Weight： _______kg Blood type：

Body mass index： [weight (Kg)/height(m)^2^] Gender：male 🞏 female 🞏

Ethnic group：

Confirmed infection (e.g., urine culture, sputum culture)：

HbA1c： % C-peptide： ng/ml

Serology negative positive Unclear or undetected

HIV 🞏 🞏 🞏

HBsAg 🞏 🞏 🞏

HbcAb 🞏 🞏 🞏

HCV 🞏 🞏 🞏

CMV 🞏 🞏 🞏

Medical history：

External chest compressions：Yes 🞏 No 🞏 date：

Hospital stays (ICU)：

Time of death： / / (Year/Month/Day) :_ (Hours: minutes)

Cause of death：

Pancreatic perfusate： Perfusate volume：

Whether the pancreas is treated before preservation：

Transport method (preservation solution)：
